# Supplementary material for: Structure of the active pharmaceutical ingredient bismuth subsalicylate
Source: Nat Commun. 2022 Apr 13;13:1984. doi: 10.1038/s41467-022-29566-0 (PMC9008038; doi:10.1038/s41467-022-29566-0)
Supplement: Supplementary file 1 — Supplementary Information [file 41467_2022_29566_MOESM1_ESM.pdf]

Supplementary Information

Structure of the active pharmaceutical ingredient bismuth subsalicylate

Authors: Erik Svensson Grape, Victoria Rooth, Mathias Nero, Tom Willhammar*, A. Ken Inge*

Affiliation:

Department of Materials and Environmental Chemistry, Stockholm University, Stockholm, 10691, Sweden. Email: tom.willhammar@mmk.su.se (T.W.); andrew.inge@mmk.su.se (A.K.I.)

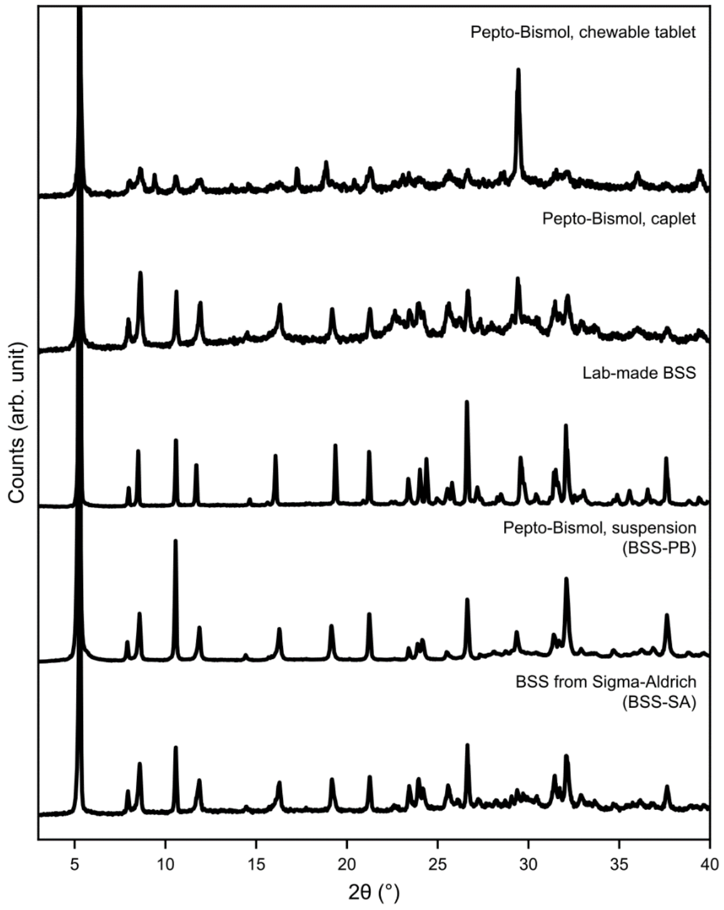

Supplementary Figure 1. Powder X-ray diffraction patterns of bismuth subsalicylate samples. Comparison of PXRD patterns acquired from bismuth subsalicylate (BSS) including lab-made BSS, BSS purchased from Sigma-Aldrich (BSS-SA), as well as various Pepto-Bismol products, including a Pepto-Bismol suspension (BSS-PB). Pepto-Bismol in chewable tablet and caplet formulations consist of additional crystalline phases such as calcium carbonate as inactive ingredients, which contribute additional reflections. Data were acquired on an in-house diffractometer (Malvern-Panalytical X'Pert Pro) set up in a Bragg-Brentano geometry, using CuKα1,2 radiation (λ_1 = 1.5406 Å, λ_2 = 1.544 Å).

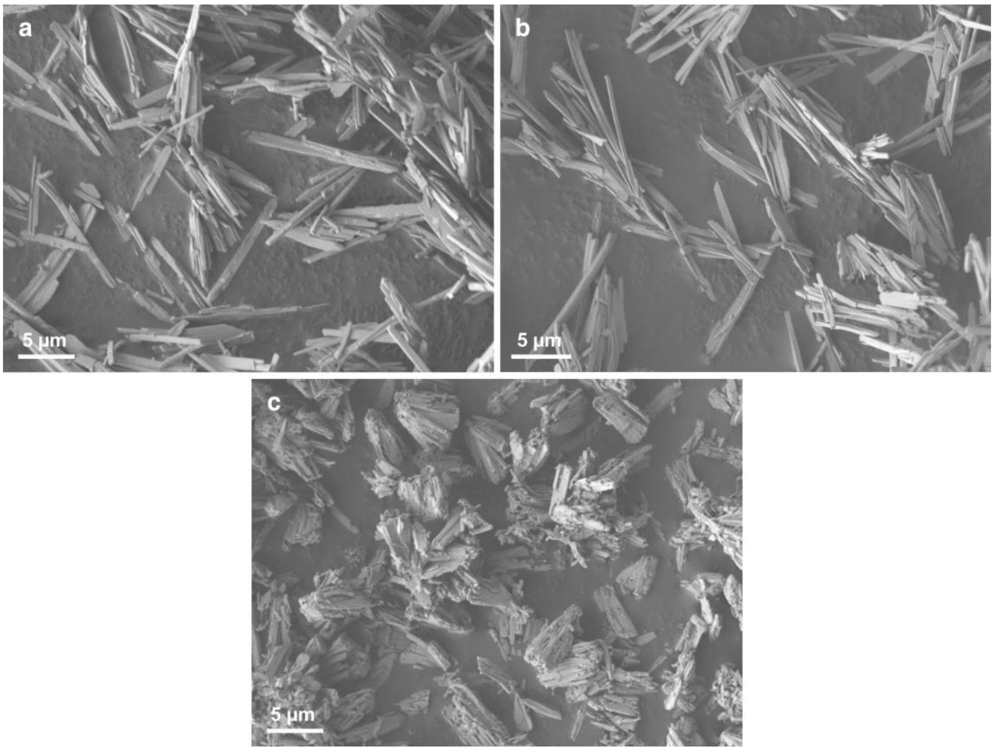

Supplementary Figure 2. Scanning electron micrographs of select bismuth subsalicylate samples. a) Commercially available bismuth subsalicylate from Sigma-Aldrich (BSS-SA). b) Commercially available bismuth subsalicylate from Sigma-Aldrich after washing with water, showing no significant changes to crystal size or morphology. c) SEM image of a Pepto-Bismol suspension (BSS-PB) after washing with water.

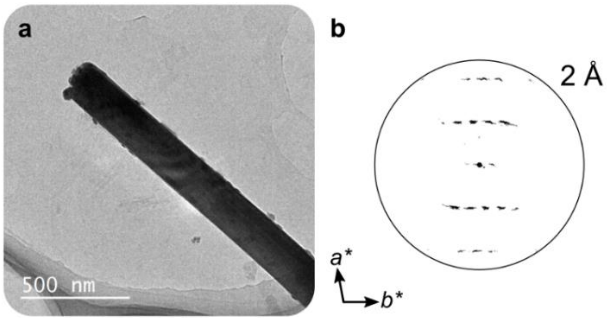

Supplementary Figure 3. Three-dimensional electron diffraction of a crystal isolated from Pepto-Bismol suspension. a) TEM image of one of the crystals studied which was washed out from a Pepto-Bismol suspension (BSS-PB). b) Reconstructed reciprocal space projection for the data acquired from the imaged crystal, as viewed along c*, showing limited scattering to a resolution of about 2 Å (drawn as a black circle) as well as ill-defined rows of reflections.

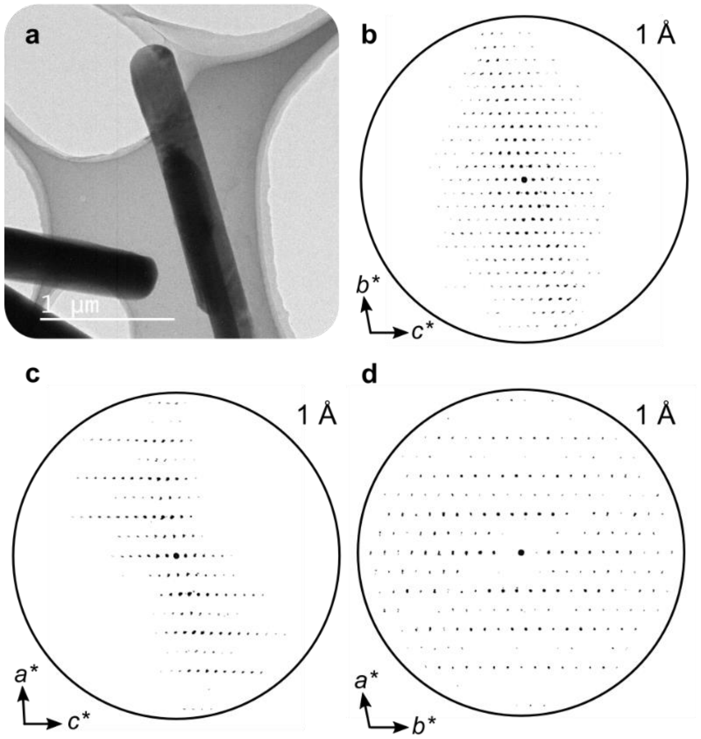

Supplementary Figure 4. Three-dimensional electron diffraction of a crystal isolated of commercially available bismuth subsalicylate. a) TEM image of one of the crystals studied from BSS acquired from Sigma-Aldrich (BSS-SA). The dataset was subsequently merged with datasets collected from 11 other crystals for structure determination. b-d) Reconstructed reciprocal space projections for the data acquired from the imaged crystal, as viewed along a*, b*, and c* (circles are drawn at a resolution of 1 Å).

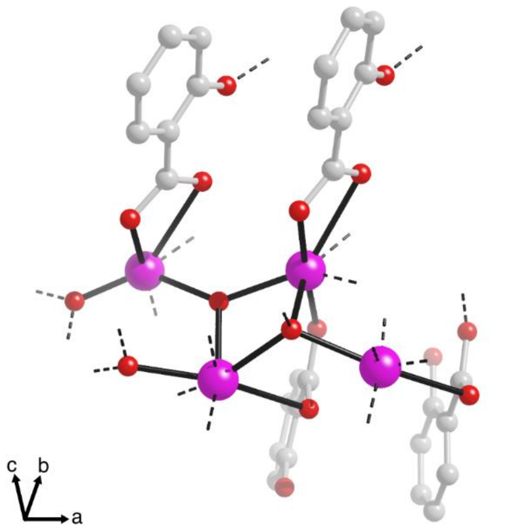

Supplementary Figure 5. Asymmetric unit for the structure of bismuth subsalicylate. Overall, the crystal structure comprises four crystallographically unique Bi^{3+} cations, four O^{2-} anions, and four salicylate ($Hsal^{-}$) anions. Dashed black lines are drawn to show bonds to symmetry-equivalent parts of the structure.

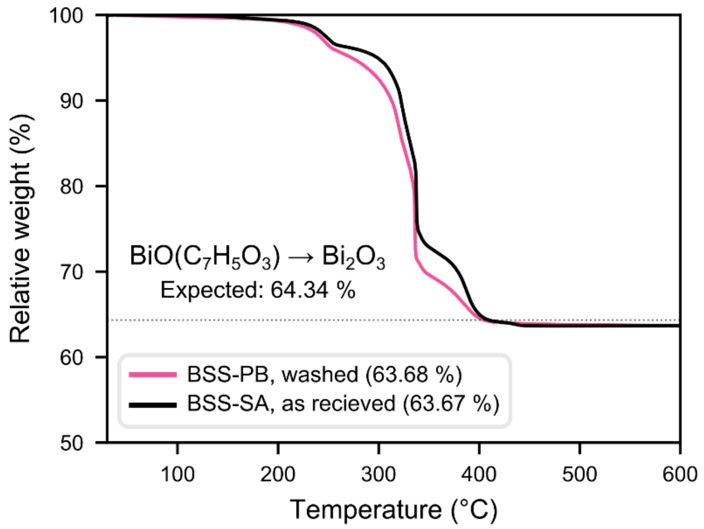

Supplementary Figure 6. Thermogravimetric analysis of commercially available bismuth subsalicylate. Thermogravimetric data acquired in air while heating bismuth subsalicylate from Sigma-Aldrich (BSS-SA) as well as washed and dried Pepto-Bismol suspension (BSS-PB).

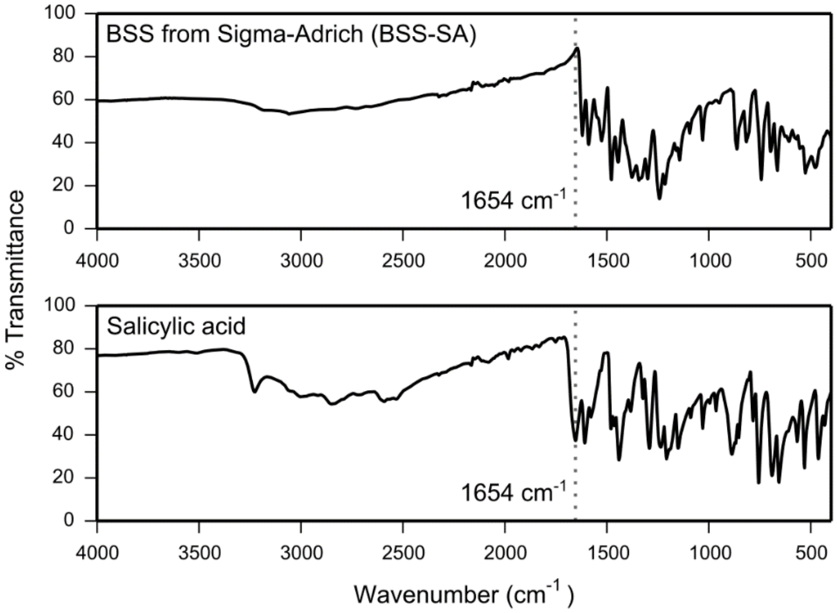

Supplementary Figure 7. FT-IR spectra of bismuth salicylate and salicylic acid. FT-IR data acquired of bismuth subsalicylate from Sigma-Aldrich (BSS-SA) as well as pure salicylic acid. Note the absence of the C=O stretching vibration expected for a protonated carboxylic acid group (1654 cm^{-1}) in the BSS-SA spectra, instead showing a band shifted to lower wavenumber, as expected for a carboxylate ion (1600 - 1560 cm^{-1}).

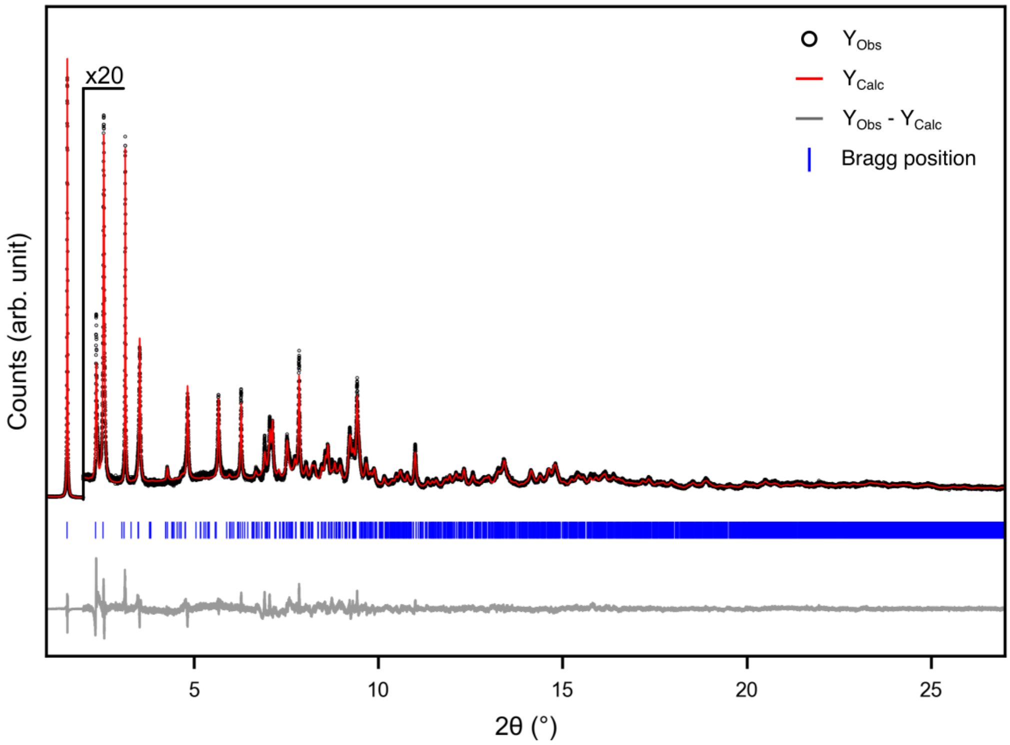

Supplementary Figure 8. Rietveld plot for the refinement of bismuth subsalicylate (BSS-PB). The plot shows the results of refining the model acquired from 3DED data against powder X-ray diffraction data collected on dried Pepto-Bismol suspension. High-resolution data were collected through the mail-in system at 11-BM, APS, Argonne National Laboratory, USA. λ = 0.458092 Å.

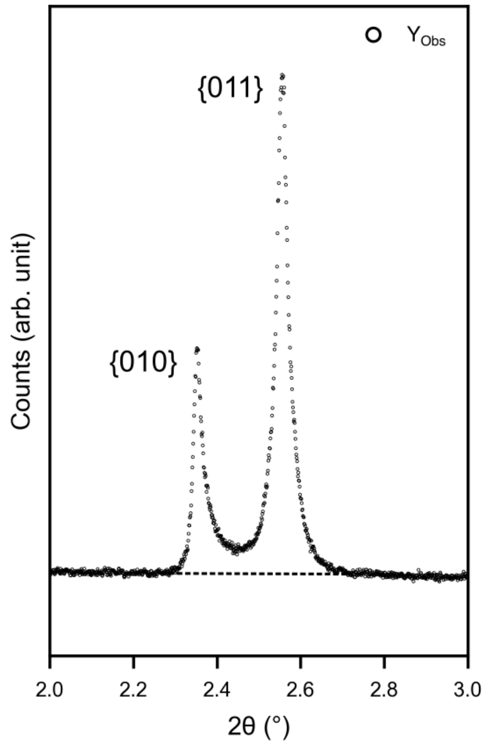

Supplementary Figure 9. Diffuse scattering observed in powder X-ray diffraction data of bismuth subsalicylate (BSS-PB). Plot showing the peaks of the {010} and {011} planes for powder X-ray diffraction data acquired from a dried Pepto-Bismol suspension, showing a significantly elevated background (dashed line as reference) as well as peak asymmetry. High-resolution data were collected through the mail-in system at 11-BM, APS, Argonne National Laboratory, USA. λ = 0.458092 Å.

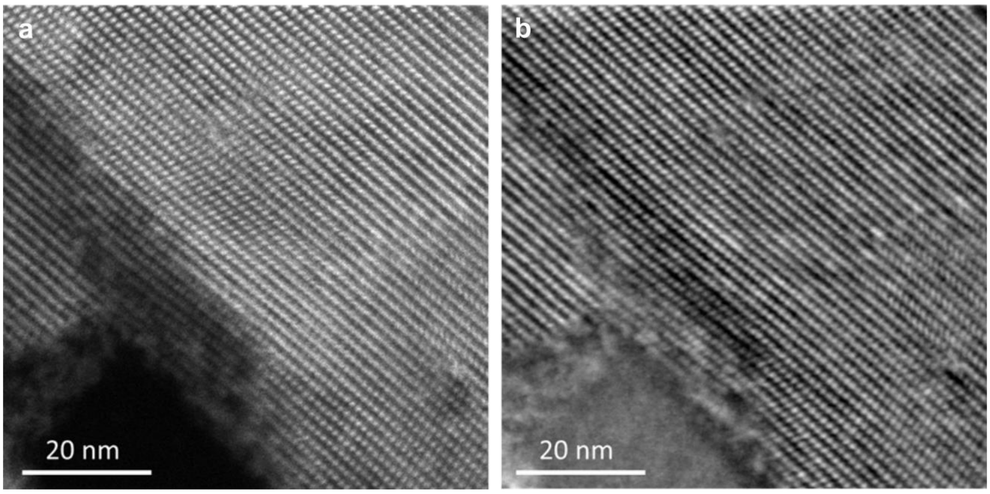

Supplementary Figure 10. Scanning transmission electron micrographs of bismuth subsalicylate crystals isolated from a Pepto-Bismol suspension (BSS-PB). a) ADF STEM image acquired along the [100] direction of a crystal. b) iDPC STEM image acquired of the same area.

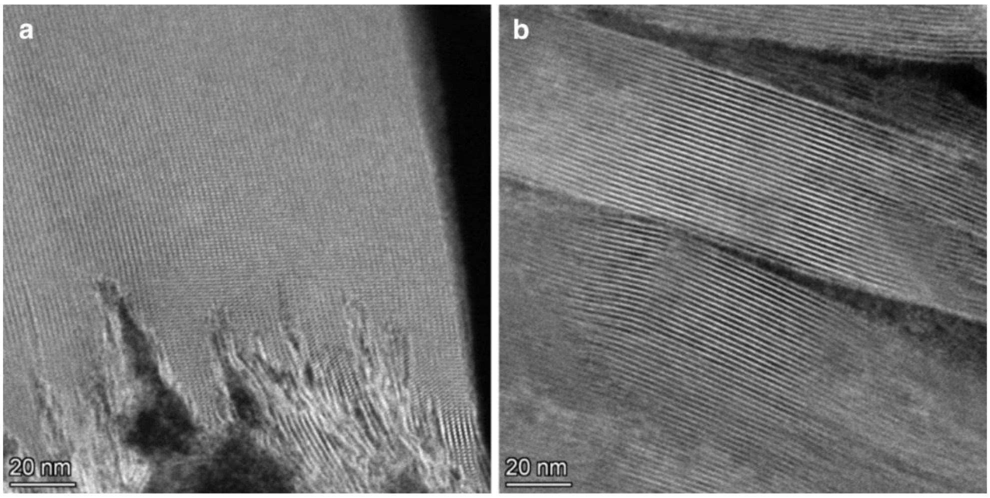

Supplementary Figure 11. Annular dark-field scanning transmission electron micrographs of bismuth subsalicylate crystals isolated from a Pepto-Bismol suspension (BSS-PB). a) ADF-STEM image from a BSS-PB crystal showing fringing of the structure close to the (010) facet. b) ADF-STEM image showing buckling and bending of the layers.

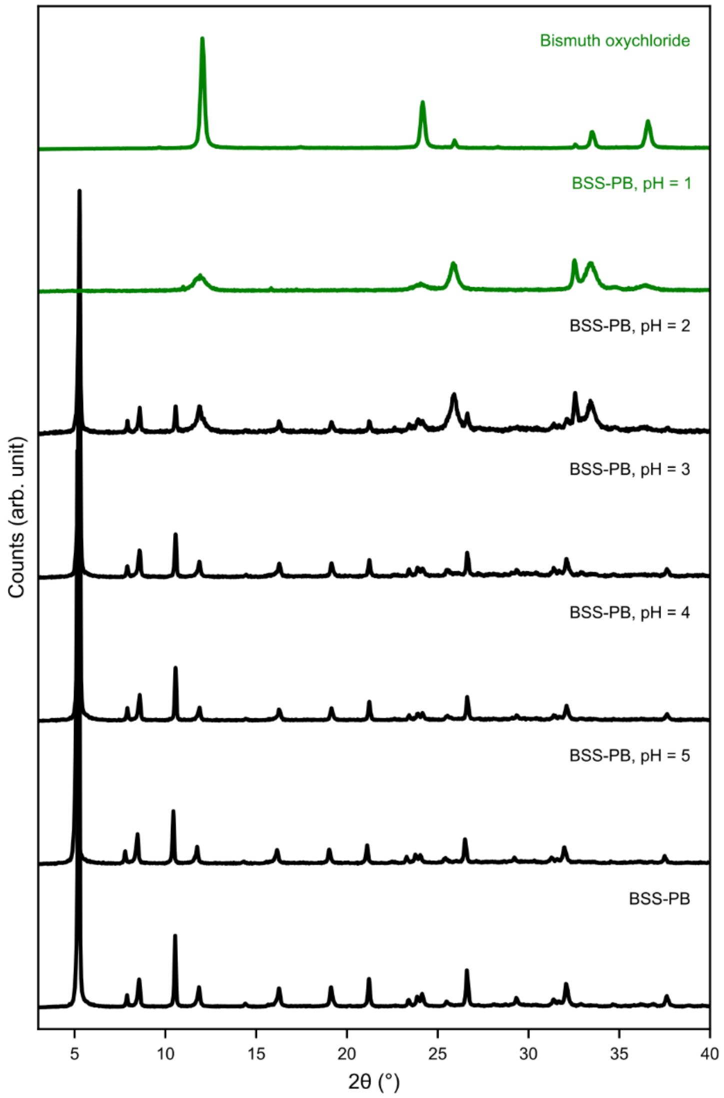

Supplementary Figure 12. Powder X-ray diffraction patterns of bismuth subsalicylate exposed to aqueous solutions of varying pH. Comparison of PXRD patterns acquired from bismuth subsalicylate (BSS-PB), isolated from a Pepto-Bismol suspension, after exposure to aqueous solutions of HCl. At low pH, the BSS is converted into bismuth oxychloride. Data were acquired on an in-house diffractometer (Malvern-Panalytical X'Pert Pro) set up in a Bragg-Brentano geometry, using CuKα1,2 radiation (λ_1 = 1.5406 Å, λ_2 = 1.544 Å).

Supplementary Table 1. Crystallographic table for 3D electron diffraction data (a merging of 12 individual datasets) collected on commercially available bismuth subsalicylate crystals from Sigma-Aldrich (BSS-SA) (CCDC deposition 2111213).

Specimen	**Bismuth subsalicylate (BSS)**
Wavelength (Å)	0.0251
Temperature (K)	96
Crystal system	Triclinic
Space group	$P\bar{1}$ (No. 2)
Unit cell dimensions	a = 8.35 Å
	b = 12.17 Å
	c = 18.09 Å
	α = 77.92°
	β = 83.16°
	γ = 76.69°
Volume (Å^3)	1744 Å^3
Z	2
Index ranges	$-10 \leq h \leq 10$
	$-15 \leq k \leq 15$
	$-20 \leq l \leq 20$
Reflections collected	60351
Independent reflections	5864
	[R(int) = 0.3724]
Completeness (to 0.8 Å resolution)	84.5 %
I/σ_I (Inf. – 0.8 Å), I/σ_I (0.9 – 0.8 Å)	5.83, 3.70
$CC_{1/2}$	0.795
R_1 (ED model) [$I > 4\sigma(I)$]	0.3043
Correlation coefficient cut-off for merged data	0.90

Supplementary Table 2. List of Bi-O distances determined from 3D electron diffraction data (a merging of 12 individual datasets) collected on commercially available bismuth subsalicylate crystals from Sigma-Aldrich (BSS-SA).

**atom 1**	**atom 2**	**distance (Å)**	**oxygen species**
Bi2	O5	2.28	carboxylate
Bi4	O11	2.50	carboxylate
Bi4	O14	2.65	carboxylate
Bi2	O8	2.72	carboxylate
Bi4	O15	2.79	carboxylate
Bi1	O12	2.80	carboxylate
Bi3	O12	2.88	carboxylate
Bi1	O8	2.95	carboxylate
Bi3	O14	2.97	carboxylate
Bi2	O9	3.01	carboxylate
Bi3	O6	3.10	carboxylate
Bi1	O6	3.14	carboxylate
Bi2	O10	3.13	phenolic
Bi4	O16	3.17	phenolic
Bi4	O10	3.23	phenolic
Bi4	O4	2.19	μ_3-O
Bi3	O4	2.19	μ_3-O
Bi1	O1	2.22	μ_3-O
Bi3	O1	2.24	μ_3-O
Bi4	O2	2.25	μ_3-O
Bi1	O2	2.26	μ_3-O
Bi2	O2	2.36	μ_3-O
Bi3	O1	2.39	μ_3-O
Bi2	O4	2.65	μ_3-O
Bi1	O3	2.33	μ_4-O
Bi2	O3	2.37	μ_4-O
Bi1	O3	2.45	μ_4-O
Bi3	O3	2.54	μ_4-O

Supplementary Table 3. Crystallographic table for the structure refinement of BSS against powder X-ray diffraction data collected on a dried Pepto-Bismol suspension (CCDC deposition 2095448).

Identification code	**Bismuth subsalicylate (BSS)**
Crystal system	Triclinic
Space group	$P\bar{1}$ (No. 2)
Unit cell dimensions	a = 8.071(6) Å
	b = 11.617(8) Å
	c = 17.42(1) Å
	α = 77.791(3)°
	β = 82.634(4)°
	γ = 82.673(5)°
Volume ($Å^3$)	1574(2) $Å^3$
Wavelength	0.458092 Å
Refinement method	Rietveld method
Refinement statistics	R_{wp} = 8.00 %
	R_{Bragg} = 1.88 %
	GOF = 1.49
